# Hémolacrymie idiopathique chez une adolescente de 15 ans

**DOI:** 10.11604/pamj.2026.54.1.49559

**Published:** 2026-05-04

**Authors:** Laatfa Imane, Youssef Jeddi

**Affiliations:** 1 Urgences Médicales Pédiatriques, Hôpital d'Enfants de Rabat, Rabat, Maroc,; 2 Faculté de Médecine et de Pharmacie de Rabat, Université Mohammed V de Rabat, Rabat, Maroc

**Keywords:** Hémolacrymie, larmes sanglantes, adolescente, idiopathique, hemolacria, bloody tears, adolescent, idiopathic

## Abstract

Hemolacria is a rare condition characterised by the discharge of bloody tears. It may be secondary to local or systemic causes, but is sometimes idiopathic. We report the case of a 15-year-old girl with no prior medical history, presenting with intermittent episodes of bilateral hemolacria for one month. Clinical, ophthalmological and ENT examinations were unremarkable. Laboratory tests, including a complete blood count and coagulation tests (prothrombin time, activated partial thromboplastin time, fibrinogen, von Willebrand factor, and platelet aggregation tests), were strictly normal, with no evidence of a hematological or coagulation disorder. Imaging (brain and orbit MRI) revealed no abnormalities. In the absence of an identified etiology, a diagnosis of idiopathic hemolacria was retained. The course was marked by persistence of episodes, without hemodynamic or systemic consequences. Idiopathic hemolacria is an exceptional clinical entity, particularly in adolescent girls, and requires regular monitoring to detect any possible underlying cause.

## Image en médecine

L'hémolacrymie est une affection rare se manifestant par l'émission de larmes sanglantes. Elle peut être secondaire à des causes locales ou systémiques, mais demeure parfois idiopathique. Nous rapportons l'observation d'une adolescente de 15 ans, sans antécédents médicaux, présentant depuis un mois des épisodes d'hémolacrymie bilatérale intermittents. L'examen clinique, ophtalmologique et oto-rhino-laryngologique était sans particularité. Le bilan biologique, comprenant la numération de la formule sanguine ainsi que les explorations de l'hémostase (taux de prothrombine, temps de céphaline activée, fibrinogène, facteur de von Willebrand et tests d'agrégation plaquettaire), était strictement normal, sans argument en faveur d'un trouble hématologique ou de la coagulation. L'imagerie (IRM cérébro-orbitaire) n'a révélé aucune anomalie. En l'absence d'étiologie identifiée, le diagnostic d'hémolacrymie idiopathique a été retenu. L'évolution a été marquée par la persistance des épisodes, sans conséquence hémodynamique ni systémique. L'hémolacrymie idiopathique constitue une entité clinique exceptionnelle, en particulier chez l'adolescente, et impose une surveillance régulière afin de dépister une éventuelle cause sous-jacente.

**Figure 1:**
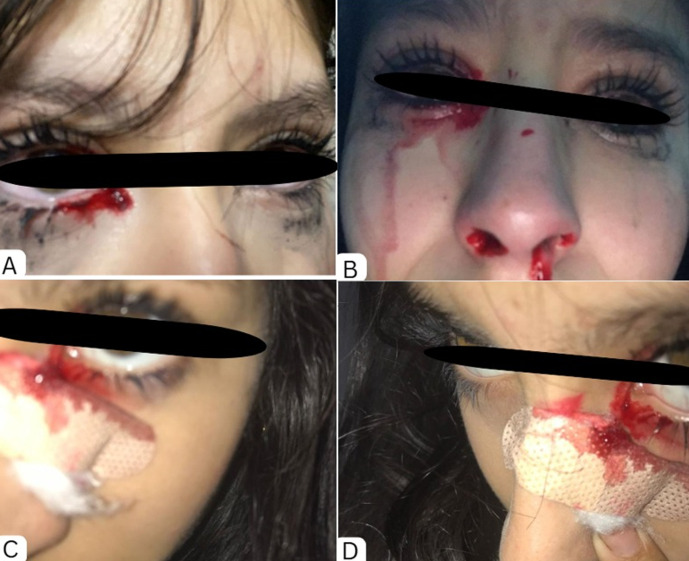
A) épisode initial d'hémolacrymie de l'œil droit; B) récidive de l'œil droit avec larmes sanglantes et écoulement nasal concomitant; C) épisode d'hémolacrymie de l'œil gauche (premier épisode); D) épisode d'hémolacrymie de l'œil gauche (récidive)

